# The implementation of natural language processing to extract index lesions from breast magnetic resonance imaging reports

**DOI:** 10.1186/s12911-019-0997-3

**Published:** 2019-12-30

**Authors:** Yi Liu, Qing Liu, Chao Han, Xiaodong Zhang, Xiaoying Wang

**Affiliations:** 1 Department of Radiology, Peking University First Hospital, No. 8 Xishiku Street, Xicheng District, Beijing, 100034 China; 2 Department of Radiolog, Peking University Cancer Hospital and Institute, No. 52 Fucheng Road, Haidian District, Beijing, China

**Keywords:** BI-RADS, Breast cancer, Index lesion, Magnetic resonance imaging, Natural language processing, Rule-based method

## Abstract

**Background:**

There are often multiple lesions in breast magnetic resonance imaging (MRI) reports and radiologists usually focus on describing the index lesion that is most crucial to clinicians in determining the management and prognosis of patients. Natural language processing (NLP) has been used for information extraction from mammography reports. However, few studies have investigated NLP in breast MRI data based on free-form text. The objective of the current study was to assess the validity of our NLP program to accurately extract index lesions and their corresponding imaging features from free-form text of breast MRI reports.

**Methods:**

This cross-sectional study examined 1633 free-form text reports of breast MRIs from 2014 to 2017. First, the NLP system was used to extract 9 features from all the lesions in the reports according to the Breast Imaging Reporting and Data System (BI-RADS) descriptors. Second, the index lesion was defined as the lesion with the largest number of imaging features. Third, we extracted the values of each imaging feature and the BI-RADS category from each index lesion. To evaluate the accuracy of our system, 478 reports were manually reviewed by two individuals. The time taken to extract data by NLP was compared with that by reviewers.

**Results:**

The NLP system extracted 889 lesions from 478 reports. The mean number of imaging features per lesion was 6.5 ± 2.1 (range: 3–9; 95% CI: 6.362–6.638). The mean number of imaging features per index lesion was 8.0 ± 1.1 (range: 5–9; 95% CI: 7.901–8.099). The NLP system demonstrated a recall of 100.0% and a precision of 99.6% for correct identification of the index lesion. The recall and precision of NLP to correctly extract the value of imaging features from the index lesions were 91.0 and 92.6%, respectively. The recall and precision for the correct identification of the BI-RADS categories were 96.6 and 94.8%, respectively. NLP generated the total results in less than 1 s, whereas the manual reviewers averaged 4.47 min and 4.56 min per report.

**Conclusions:**

Our NLP method successfully extracted the index lesion and its corresponding information from free-form text.

## Background

Breast cancer is one of the most commonly diagnosed types of cancer and a major global cause of cancer mortality in women, accounting for 23% of cancer diagnoses and 14% of cancer mortality each year [1]. Breast MRI can detect clinically and mammographically occult breast cancer in 3.1% of women with newly diagnosed breast cancer [2–5]. In 2007, the American Cancer Society issued a new clinical guideline recommending annual MRI screening for high-risk women [6–9].

Definitions of imaging features of breast MRI reports are clearly outlined in the Breast Imaging Reporting and Data System (BI-RADS) lexicon by the American College of Radiology [10]. BI-RADS descriptors have been used by radiologists in our department to describe breast lesion features and impression in breast MRI reports since 2013. This reduces variation between radiologists and standardizes the vocabulary used in MRI reports.

There are often multiple lesions in breast MRI reports and radiologists usually focus on describing the index lesion that is most crucial to clinicians in determining the management and prognosis of patients. Thus, identifying and extracting the index lesion is a critical clinical task. We assumed that the index lesion accounts for the largest number of imaging features and tried to extract the index lesions from free-form text by radiologists. While manual review may be feasible for small-scale studies, this method is very time-consuming, error-prone, and costly in studies with large sample sizes [11].

Natural language processing (NLP) consists of multiple steps, including a computer-based approach that converts free-form text into a standardized structured format with the help of lexicons and ontologies, ultimately creating standardized and normalized concepts. The structured information from reports could be acquired by NLP to determine index lesions, assuming all pertinent imaging features are recorded in the report [12]. NLP has been used for breast cancer diagnosis previously, including the extraction of BI-RADS categories and lesions from mammography reports [13, 14]. However, few studies have investigated NLP in breast MRI data based on free-form text. The objective of the current study was to assess the validity of our NLP program to accurately extract index lesions and their corresponding imaging features from free-form text of breast MRI reports using the rule-based method.

## Methods

### Data

This was a retrospective study that received approval from the responsible institutional review board of Peking University First Hospital with waiver of informed consent (2016[1178]). All breast MRI reports (*n* = 1633) which were written in Chinese from 2014 to 2017 were collected from Peking University First Hospital. 1633 patients were recruited into the study and each patient corresponded to one report. We used 1160 of the 1633 reports for development and refinement of the NLP system; the 478 not used for developing the system were selected and held out as an independent test set for the final evaluation of the NLP system. All patients met the following inclusion criteria: examination type was identified correctly and images were available for evaluation (T1- and T2-weighted fast spin-echo, diffusion-weighted imaging, echo planar imaging, short time inversion recovery, and dynamic contrast-enhanced T1-weighted fast spin-echo). Postoperative cases were excluded.

### BI-RADS MRI lexicon

All imaging features and values were predefined based on the BI-RADS MRI lexicon [15] by two experienced experts in the field of breast imaging (Table 1). We also used RadLex, a controlled lexicon for radiology terminology, to identify semantic classes for terms in radiology reports. Our information extraction task focused on recognizing two semantic types of named entities: imaging features and their values. Imaging features are terms that refer to abnormalities in the breast and its attributes, such as mass, anatomic locations, size, shape, margin, signal, and enhancement kinetics. Imaging values are terms that modify imaging features to describe their features, f.e. “*upper outer quadrant of the right breast*” describes the location of a mass in the breast. BI-RADS categories include 0, 1, 2, 3, 4, 5, and 6 (Table 2).

**Table 1:** Imaging features and their value set to be extracted from breast MRI reports

Entity type	Imaging features	Data type	Value set of imaging feature	Units
Mass	Location	Categorical	Right, Left, Upper, Outer quadrant …	N/A
	Shape	Categorical	Oval, Round, Irregular …	N/A
	Size	Numerical		mm
	Signal	Categorical		N/A
	T1WI	Categorical	Low signal, Isointensity, High signal	N/A
	T2WI	Categorical	Low signal, Isointensity, High signal	N/A
	DWI	Categorical	Low signal, Isointensity, High signal	N/A
	Margin	Categorical	Smooth, irregular, spiculated...	N/A
	Internal enhancement	Categorical	Homogeneous, Heterogeneous …	N/A
	Enhancement kinetic curve	Categorical	Persistent, Plateau, Wash-out	N/A
NME	Location	Categorical	Right, Left, Upper, Outer quadrant …	N/A
	Distribution pattern	Categorical	Focal area, Linear, Segmental …	N/A
	Scope	Numerical		mm
	Signal	Categorical		N/A
	T1WI	Categorical	Low signal, Isointensity, High signal	N/A
	T2WI	Categorical	Low signal, Isointensity, High signal	N/A
	DWI	Categorical	Low signal, Isointensity, High signal	N/A
	Internal enhancement	Categorical	Homogeneous, Heterogeneous...	N/A
	Enhancement kinetic curve	Categorical	Persistent, Plateau, Wash-out	N/A
Other associated findings	Lymphadenopathy	Categorical		N/A
	Invasion of skin, nipple, chest wall, pectoralis muscle	Categorical		N/A

*T1WI* T1-weighted imaging, *T2WI* T2-weighted imaging, *DWI* diffusion weighted imaging, *NME* non-mass enhancement, *N/A* not applicable

**Table 2:** BI-RADS assessment categories and the number of index lesions found for each category

BI-RADS category	Implication	Number
0	Requires additional imaging assessment and/or prior imaging for comparison	0
1	Negative	0
2	Benign discovery	28
3	Probably benign discovery	60
4	Suspected suspicious abnormality - biopsy	87
5	Highly suggestive of malignancy - appropriate action should be taken	119
6	Malignancy confirmed by biopsy - clinically feasible surgical resection	177

*BI-RADS* Breast Imaging Reporting and Data System

### Preprocessing

**Fig. 1:**
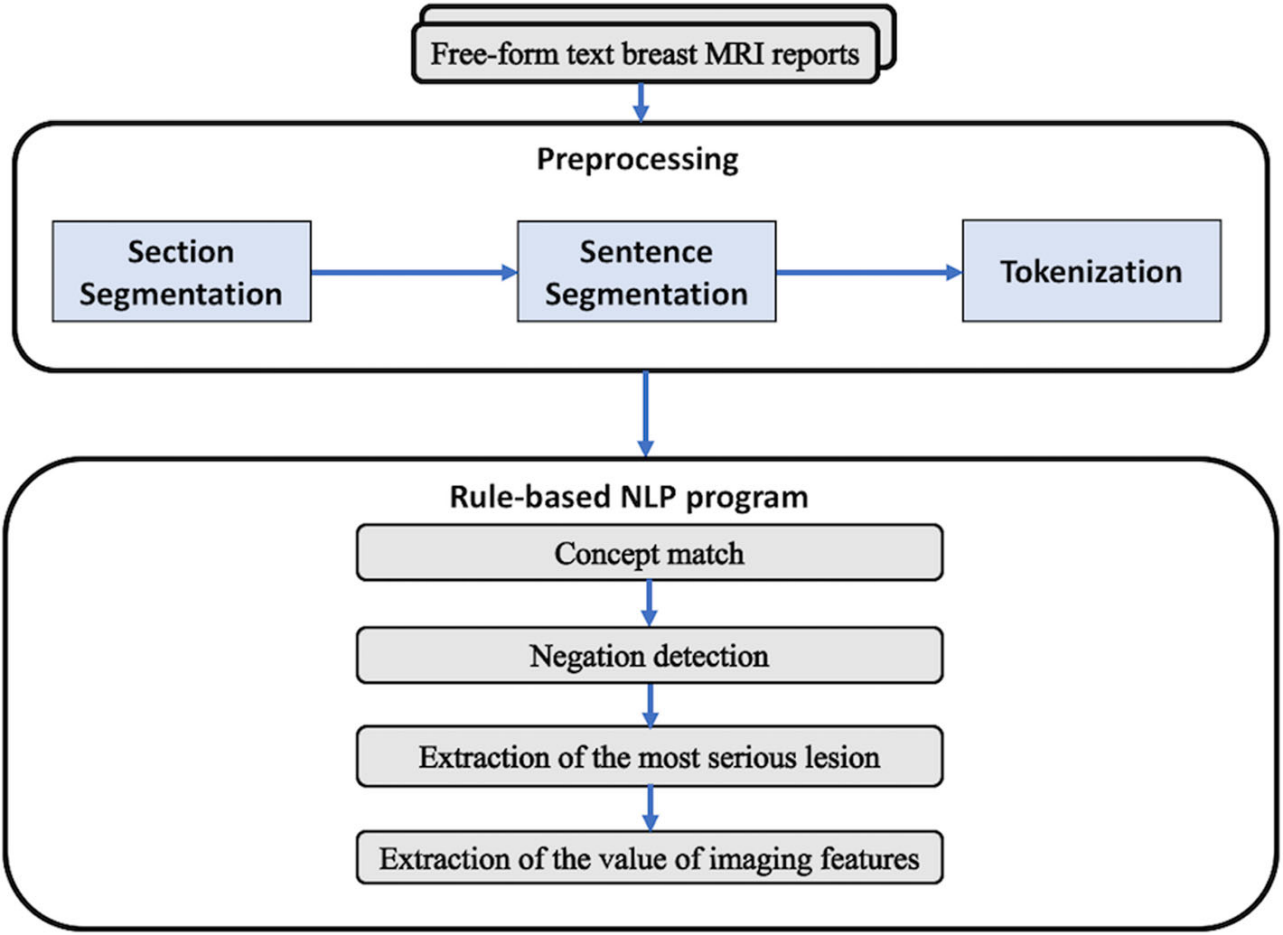
An overview of our NLP program for extracting breast MRI reports

**Fig. 2:**
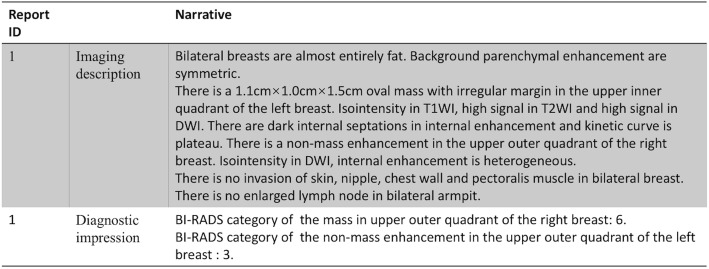
A representative original breast MRI report. The report consists of an imaging description and diagnostic impression

**Fig. 3:**
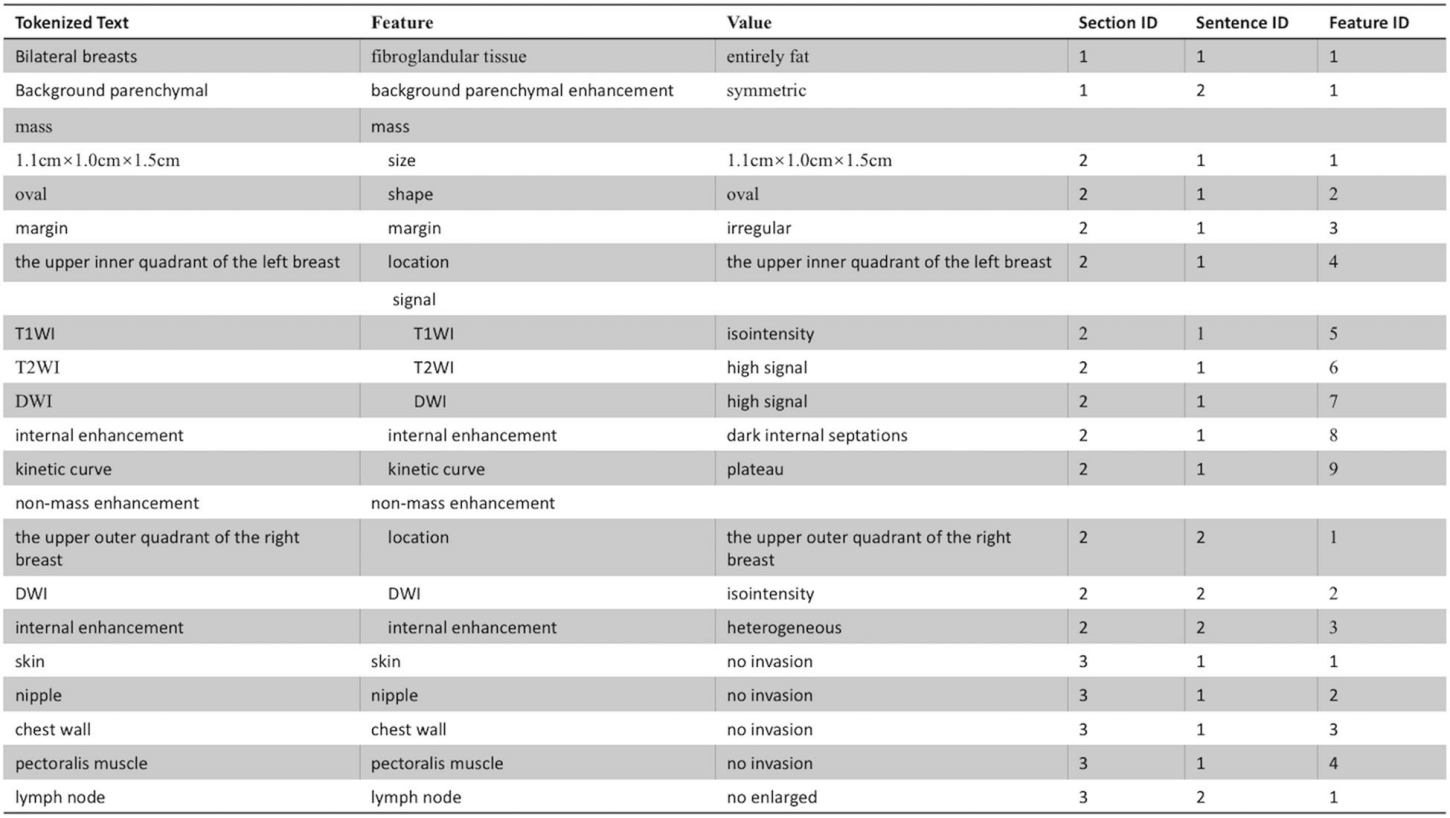
Annotated text with the final extraction results. Each report was converted into a list of vocabulary flagged with its section, sentence, and vocabulary number after section segmentation, sentence segmentation, and tokenization

An internally developed NLP program (Smartree Clinical Information System, Beijing) was used to detect the index lesion and imaging features for each imaged breast from text reports (Fig. 1). This internally developed NLP program contained several analysis engines for various linguistics and clinical tasks, such as section segmentation, sentence segmentation and detection, tokenization, concept detection and normalization. In our pipeline, sequential processing modules were executed, producing an output of structured information of the index lesion. As shown in Fig. 2, each original breast MRI report spanned multiple paragraphs and occasionally split at various locations. The steps were as previously reported [16]. To facilitate NLP analysis, text reports were preprocessed. First, section boundaries were noted. This preprocessing segment input the original breast MRI report into sections. In the original breast MRI report, each section began with an anatomical location that was followed by another anatomical location (Example 1). Section segmentation was implemented as a predefined grammar consisting of a set of phases that annotate the section headers, that was, the anatomical location. Second, the sentence boundaries were noted. Sentence segmentation was implemented using the predefined rule that used the periods as the marker word (Example 2). Third, our NLP program preprocessed the reports for imaging features tokenization based on our dictionary (Example 3). We developed the list of synonyms by inspecting a set of other 500 reports in addition to the predefined BI-RADS MRI lexicon. In addition, spelling mistakes were corrected and abbreviations were expanded to their full form. As shown in Figure 3, each breast MRI report was converted into a list of vocabulary.

#### Example 1:

Section segmentation.

There is a 1.1 cm × 1.0 cm × 1.5 cm oval mass with irregular margin in [the upper inner quadrant of the left breast] ***location***. Isointensity in T1WI, high signal in T2WI and high signal in DWI. There are dark internal septations in internal enhancement and kinetic curve is plateau. There is a non-mass enhancement in [the upper outer quadrant of the right breast] ***location***. Isointensity in DWI, internal enhancement is heterogeneous.

#### Example 2:

Sentence segmentation.

There is a 1.1 cm × 1.0 cm × 1.5 cm oval mass with irregular margin in the upper inner quadrant of the left breast [.] ***period*** Isointensity in T1WI, high signal in T2WI and high signal in DWI[.] ***period*** There are dark internal septations in internal enhancement and kinetic curve is plateau [.] ***period.***

#### Example 3:

Synonym conversion.

There is a 1.6 cm × 1.2 cm × 1.2 cm oval [mass] ***synonym: lesion*** with [smooth] ***synonym: clear*** boundary in the upper inner quadrant of the left breast.

Defining the index lesion by the number of imaging features.

The next step was concept match. Our processing module could match input features to BI-RADS terms by both exact match and synonym match. Then, we took the negation detection step. This step was to check the negated concepts. Distance and direction was both used to restrict the association between negation words and corresponding features. Three-word rule was imposed as the default distance for negative words after reviewing other 500 reports. We extracted negative words based on the given rule using our NLP tool.

We developed a strategy to compare the number of imaging features in each lesion, assuming that the index lesion accounts for the largest number of imaging features. We also selected the corresponding BI-RADS categories from which the highest score of each case was stored.

Extracting the value of imaging features from index lesions.

The last step was to extract the value of imaging features from index lesions and the accompanying signs (e.g. surrounding invasion or lymph node) as well as the highest BI-RADS category as the final BI-RADS category for the index lesion. The final results are displayed in Fig. 3.

### Standard reference

In order to evaluate the accuracy of our NLP system, 478 selected reports were reviewed by two diagnostic radiologists with 2 and 6 years of postgraduate experience. Two manual reviewers were blinded from the NLP results and extracted information independently. Any discrepancies between data found were evaluated by a third independent reviewer. Reports contained either no lesion (*n* = 7), one lesion (*n* = 93), or multiple lesions (*n* = 378). The diagnostic impression of the 7 reports with no lesion was BI-RADS 1. The remaining 471 positive cases exhibited a total of 889 lesions containing 471 index lesions. The number of index lesions for each category is displayed in Table 2. The mean number of imaging features in an index lesion was 8.2 ± 1.2 (range: 5–9; 95% confidence interval (CI): 8.092–8.308). This dataset of 478 reports comprised the gold standard for our evaluation and comparative analyses were performed.

### Time consumption

Each reviewer was asked to record the time required to extract the index lesion, corresponding imaging features, values, accompanying signs, and BI-RADS category from each report. Afterwards, we compared the time to data acquisition between the NLP program and the manual review.

### Statistical analysis

The extraction and retrieval of information by the NLP program was compared to standard reference. The kappa coefficient was used to assess the agreement between two reviewers. Good inter-observer agreement was noted between two readers when k ≥ 0.60. The mean number of imaging features per lesion and the mean number of imaging features per index lesion were described in the form of mean ± standard deviation. To calculate overall performance metrics, results were combined into the 2*2 table. Recall and precision was determined for the information collected by the NLP program, compared against the standard reference.

## Results

### Inter-observer agreement

In regard to the total number of lesions and index lesions, location, enhancement kinetic curve, nipple invasion, skin invasion, chest wall invasion, pectoralis muscle invasion and BI-RADS category of the index lesions, the inter-observer reliability showed almost perfect agreement, with k values of 0.92, 0.94, 0.83, 0.90, 0.89, 0.88, 0.90. 0.91 and 0.93, respectively. In regard to the shape, size, T1WI, T2WI, DWI and internal enhancement of the index lesions, there was substantial agreement, with a k value of 0.73, 0.78, 0.80, 0.79, 0.79 and 0.78, respectively. Any discrepancies between data found were evaluated by a third independent reviewer.

### Imaging features of lesions

The NLP system extracted 889 lesions from 478 reports. Reports contained either no lesion (*n* = 7), one lesion (*n* = 93), two lesions (*n* = 338) or three lesions (*n* = 40).

**Fig. 4:**
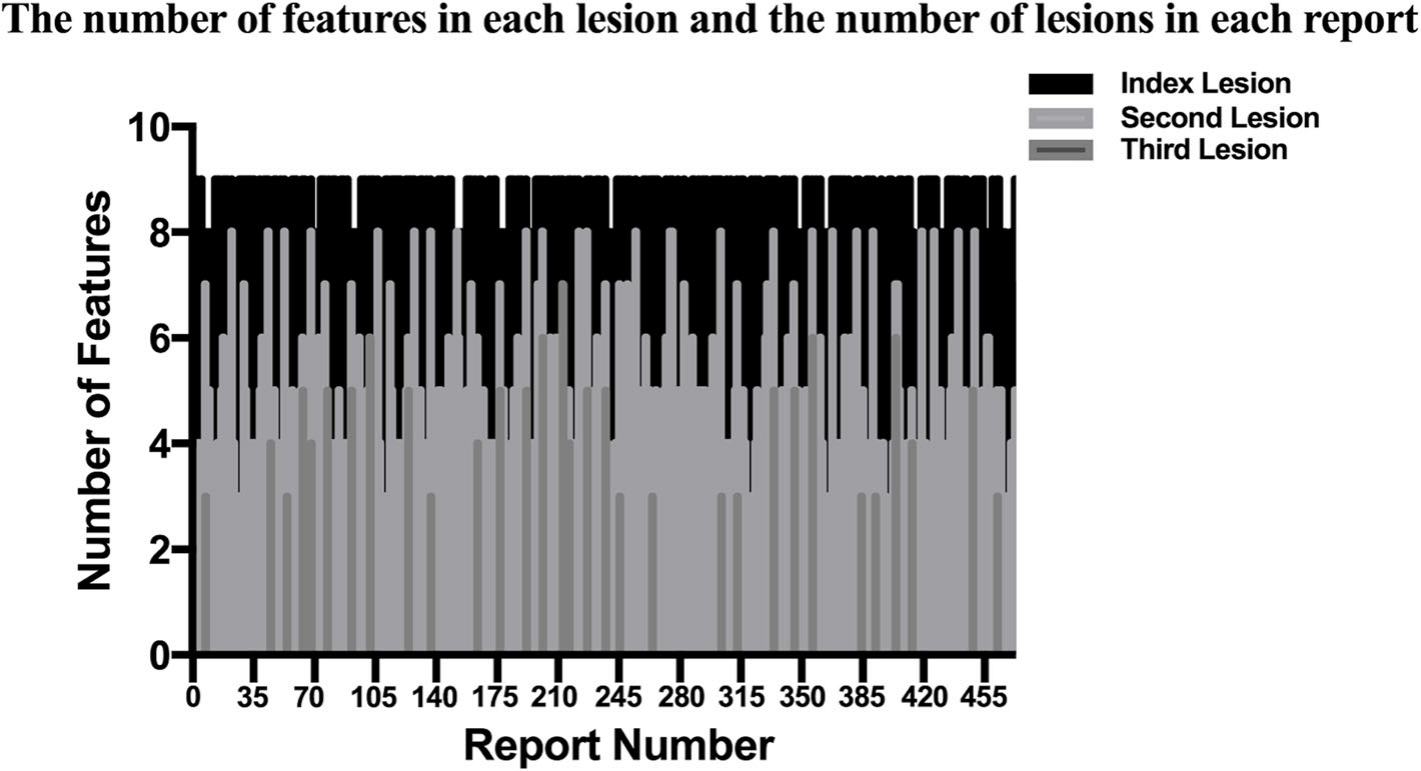
The number of features in each lesion and the number of lesions in each report

The mean number of imaging features per lesion was 7.6 ± 1.4 (range: 5–9; 95% CI: 7.315–7.885) for the reports with one lesion. For the reports with 2 lesions, the mean number of imaging features per lesion of index lesion was 8.2 ± 1.0 (range: 5–9; 95% CI: 8.093–8.307), and that of non-index lesion was 4.5 ± 1.3 (range: 3–8; 95% CI: 4.361–4.639). For the reports with 3 lesions, the mean number of imaging features per lesion of index lesion was 8.5 ± 0.7 (range: 6–9; 95% CI: 8.283–8.717), and that of non-index lesion was 5.2 ± 1.6 (range: 3–8; 95% CI: 4.849–5.551). The mean number of imaging features per lesion was 6.5 ± 2.1 (range: 3–9; 95% CI: 6.362–6.638) for all lesions and that was 8.0 ± 1.1 (range: 5–9; 95% CI: 7.901–8.099) for index lesions extracted by the NLP system. The number of features in each lesion was different. The number of features in each lesion and the number of lesions in each report was displayed in Fig. 4.

### NLP system performance during index lesion extraction

The NLP system extracted 471 index lesions and 469 of them were true. The NLP system demonstrated a recall of 99.6% and a precision of 99.6% for correct identification of index lesions.

### NLP system performance during extraction of the value of imaging features

The number of incorrectly extracted cases in each imaging feature was showed in Table 3. For all breast MRI reports, the NLP system demonstrated a recall of 91.0% and a precision of 92.6% for the correct identification of the value of imaging features from the index lesions. The recall and precision for the correct identification of the BI-RADS categories were 96.6 and 94.8%, respectively. The performance of the NLP system for individual study types is displayed in Table 4.

**Table 3:** Error analysis of the NLP system

	Imaging features	Not detected (cases)	Not detected correctly (cases)
Index lesion	Location	16	2
	Shape	2	24
	Size	20	5
	T1WI	21	–
	T2WI	18	–
	DWI	12	10
	Margin	7	9
	Internal enhancement	20	3
	Enhancement kinetic curve	20	–
	Lymphadenopathy	12	20
	Nipple invasion	29	–
	Skin invasion	43	–
	Chest wall invasion	5	–
	Pectoralis muscle invasion	3	–
	BI-RADS category	20	4

*NLP* natural language processing, *T1WI* T1-weighted imaging, *T2WI* T2-weighted imaging, *DWI* diffusion weighted imaging, *NME* non-mass enhancement, *BI-RADS* Breast Imaging Reporting and Data System

**Table 4:** Accuracy in extracting complete descriptions of breast lesions by the NLP system

Entity type	Imaging features	Recall	Precision
Mass	Location	90.1%	95.7%
	Shape	85.4%	94.1%
	Size	90.4%	94.2%
	T1WI	90.3%	94.1%
	T2WI	89.6%	94.1%
	DWI	88.7%	93.2%
	Margin	90.9%	95.9%
	Internal enhancement	91.6%	90.2%
	Enhancement kinetic curve	91.6%	95.7%
NME	Location	90.9%	95.2%
	Distribution pattern	86.2%	94.1%
	Scope	89.2%	92.3%
	T1WI	91.1%	93.6%
	T2WI	88.9%	93.2%
	DWI	88.6%	94.0%
	Internal enhancement	91.3%	91.5%
	Enhancement kinetic curve	90.9%	95.0%
Lymphadenopathy		98.7%	87.7%
Invasion	Nipple	98.6%	86.4%
	Skin	97.4%	86.8%
	Chest wall	97.7%	86.9%
	Pectoralis muscle	96.2%	85.8%
BI-RADS category		96.6%	94.8%
Overall		91.5%	92.9%

*NLP* natural language processing, *T1WI* T1-weighted imaging, *T2WI* T2-weighted imaging, *DWI* diffusion weighted imaging, *NME* non-mass enhancement, *BI-RADS* Breast Imaging Reporting and Data System

### Efficiency

We compared the time to data acquisition between the NLP program and manual review. The average time for each manual reviewer to extract the index lesion and the corresponding features was 4.47 ± 0.85 min (95% CI: 4.393–4.547) and 4.56 ± 0.89 min (95% CI: 4.480–4.640) per report, respectively, whereas NLP generated the total results in less than 1 s.

## Discussion

This present study was conducted in order to evaluate the implementation of our NLP program, which extracts index lesions and corresponding values of imaging features from MRI reports. Our results indicate that NLP can accurately identify the index lesion in each case. The recall and precision of our system for achieving perfect image information extraction were above 85.0%.

Previous studies have been published on extracting information using various NLP systems [13, 16]. For example, Gao et al. (2015) developed a rule-based NLP system to extract four mammographic findings (mass, calcification, asymmetry, and architectural distortion), which are closely related to the increased risk of breast cancer [16]. Jain et al. (1997) developed an NLP system (MedLEE) to encode the clinical information in mammogram reports to identify the suspected breast cancer [17]. Based on MedLEE, Sevenster et al. (2012) developed another NLP system, which could extract and correlate the findings and location [18]. However, those NLP system sometimes failed to extract the location, although the location phrases were added to the vocabulary. In addition, those studies used an NLP system to extract information from mammography reports, whereas few studies have addressed features/information extraction from breast MRI reports. Breast MRI is highly sensitive for detection and characterization of malignancy due to its great soft-tissue contrast capabilities. Index lesions are the most crucial factors, as they directly determine treatment and prognosis. Many retrospective studies require MRI information extracted from index lesions to analyze the association between lesion characteristics and clinical outcomes [19, 20].

Our approach utilized a rule-based method that identified index lesions and corresponding features from breast MRI reports. Our rule-based system used concept match and negation detection that mapped imaging features in the free-form text reports to respective classes and achieved extremely high performance. According to our rule that index lesions account for the largest number of imaging features, the NLP automatically selected the index lesion from each report for a variety of efforts including follow-ups for breast cancer, targeted therapy, and lesion annotation. For example, the 10-year survival rate for breast cancer exceeded 70% and the survival rate for local lesions was about 89% [21]. Therefore, follow-ups are very common for breast cancer patients. The purpose of follow-ups is to detect changes in index lesions in the short-term or assess the effects of treatment. If the NLP system is able to precisely extract and even compare the difference in index lesions at follow-ups, it will greatly eliminate the time-consuming process of manual extraction.

For all breast MRI reports in this study, NLP systems demonstrated a recall of 91.0% and a precision of 92.6% for the correct identification of the value of imaging features from the index lesions. The recall and precision for the correct identification of the BI-RADS categories were 96.6 and 94.8%, respectively. The efficiency of the extraction of the value of each imaging feature was different. In addition to “Location”, “Margin”, and “Enhancement kinetic curve”, the precision and recall of the values of other features were above 95 and 90%, respectively, which is similar to the results of previous studies on extraction of image report information [22, 23]. The reasons for extraction errors included features were not detected or they were not detected correctly by the NLP tools. The main error was that features were not detected by our tools. Most of that errors resulted from the poor compliance with the BI-RADS lexicon. Our NLP system extracted features and values of features based on the BI-RADS lexicon. Although radiologists in our department were asked to describe breast lesion features and impression using BI-RADS descriptors after 2013, not all radiologists necessarily comply to these standards, which hinders the application of NLP. For example, as to the description of lesions, various expressions such as “*a low-enhancement zone of necrosis in the center and rim homogeneous enhancement in the surrounding area*” appear in the report; however NLP system cannot extract this as “*rim enhancement*” or “*homogeneous enhancement*”. Another reason was that there were some spelling mistakes in our reports. Although spelling corrections were conducted during data preprocessing, our dictionary was not comprehensive and some infrequent spelling mistakes were not included. For example, the word “*Bi-RAS*” was used in the imaging diagnosis: “*Bi-RAS category of the mass in the upper inner quadrant of the right breast: 6*”. The NLP system could not discern that “*BI-RADS*” was “*6*”. In clinical practice, incomplete or incorrect extraction information will affect the further analysis and utilization of clinical data. Error analysis showed that the extraction errors would be significantly reduced by augmenting our dictionary in the future.

Although the inter-observer variability of manual extraction was high, there were some discrepancies between two reviewers during information extraction. Our approach utilized a rule-based method that identified index lesions and corresponding features from breast MRI reports. As long as the ruled were clearly defined, key information could be accurately extracted from the free-form text reports. Our rule-based NLP method could efficiently avoid the discrepancies between reviewers during the manual extraction process, which was conductive to the mining of free-form text reports in clinical practice.

One of the most notable advantages of NLP was that it could complete all information extraction in a matter of seconds compared to the dozens of hours it would take for the manual reviewer to obtain the same information. This difference in time becomes more apparent when dealing with an increasing number of cases, and manual reviewers with time are prone to fatigue and therefore reduced efficiency and accuracy. The NLP program could thus significantly reduce the time and achieve this with high accuracy.

The literature suggests that health information technology could improve the efficiency and quality of health care [24], and NLP is an informatics tool that can help fulfill this promise. More specifically, the extracted information from the NLP system could be used to build cohorts for cancer studies [25-27]. To date, building cohorts for cancer studies has relied on the laborious and time-consuming manual selection of cancer cases. By extracting features of cancer automatically with the NLP system, the efficiency of cancer research could be improved. Some studies have selected patients with various conditions, including renal cysts [25], adrenal nodules [26], or specific BI-RADS assessment categories [27]. In addition to extracting the BI-RADS categories, our study also extracted lesion features and the value of each feature from breast MRI reports. Furthermore, in recent years, much artificial intelligence or computer-aided diagnostic research used the information of reports to label lesions or organs. The annotation of imaging features is important and the NLP system facilitates the extraction of imaging features that can be directly used to label lesions or organs. Forsberg et al. (2017) used the clinically provided spine label annotations stored in an institution image archive as training data for deep learning-based vertebral detection [28]. The results demonstrated that clinically annotated image data from one image archive is sufficient to train a deep learning-based pipeline for accurate detection and labeling of MR images depicting the spine. Similarly, it is feasible to use annotated image data to train other disease prediction models, and simultaneous application of the NLP system to automatically extract annotation data will greatly increase the efficiency of this process.

There are some limitations to our NLP system, such as the selection of reports that are predefined for index lesions. First, the subject of our study were breast MRI free-form text reports using BI-RADS descriptors. Although our NLP system performed well on these reports, it was not suitable for other reports that did not use BI-RADS descriptors. Second, only 7 out of 478 cases reported no lesion in our data and there is no case with BI-RADS 0 or 1 in the rest of 471 reports which reflected the general population who underwent breast MRI. However, the number of negative cases was too small in our study which might impact on the method performance. We would add more negative cases to verify the performance of our NLP method in the future. In addition, the NLP system extracted the index lesion based on our hypothesis that the index lesion accounts for the largest number of imaging features. This hypothesis was artificially defined and was not the direct extraction of the index lesion. In the future, the index lesions should be directly extracted from images based on the image features.

## Conclusions

The imaging features of index lesions in breast MRI can be extracted using our NLP system, which can be used to build cohorts for cancer studies and label data automatically for model training. In the future, data from structured reports could be used for NLP research and the efficiency of NLP could be further improved with enhancements of data quality.

## Abbreviations

BI-RADS: Breast Imaging Reporting and Data System
MRI: magnetic resonance imaging
NLP: natural language processing
